# Meta-analysis of senescent cell secretomes to identify common and specific features of the different senescent phenotypes: a tool for developing new senotherapeutics

**DOI:** 10.1186/s12964-023-01280-4

**Published:** 2023-09-28

**Authors:** Yo Oguma, Nicola Alessio, Domenico Aprile, Mari Dezawa, Gianfranco Peluso, Giovanni Di Bernardo, Umberto Galderisi

**Affiliations:** 1 Department of Stem Cell Biology and Histology, Tohoku University Graduate School of Medicine, Sendai, Japan; 2 Department of Experimental Medicine, Luigi Vanvitelli Campania University, Naples, Italy; 3 International Medical University UNICAMILLUS, Rome, Italy; 4 Genome and Stem Cell Center (GENKÖK), Erciyes University, Kayseri, Turkey; 5 Center for Biotechnology, Sbarro Institute for Cancer Research and Molecular Medicine, Temple University, Philadelphia, PA USA; 6 Dip. Medicina Sperimentale, Via Luigi De Crecchio 7, 80138 Naples, Italy

**Keywords:** Senescence, Secretome, SASP, Meta-analysis

## Abstract

DNA damage resulting from genotoxic injury can initiate cellular senescence, a state characterized by alterations in cellular metabolism, lysosomal activity, and the secretion of factors collectively known as the senescence-associated secretory phenotype (SASP). Senescence can have beneficial effects on our bodies, such as anti-cancer properties, wound healing, and tissue development, which are attributed to the SASP produced by senescent cells in their intermediate stages. However, senescence can also promote cancer and aging, primarily due to the pro-inflammatory activity of SASP.

Studying senescence is complex due to various factors involved. Genotoxic stimuli cause random damage to cellular macromolecules, leading to variations in the senescent phenotype from cell to cell, despite a shared program. Furthermore, senescence is a dynamic process that cannot be analyzed as a static endpoint, adding further complexity.

Investigating SASP is particularly intriguing as it reveals how a senescence process triggered in a few cells can spread to many others, resulting in either positive or negative consequences for health. In our study, we conducted a meta-analysis of the protein content of SASP obtained from different research groups, including our own. We categorized the collected omic data based on: i) cell type, ii) harmful agent, and iii) senescence stage (early and late senescence).

By employing Gene Ontology and Network analysis on the omic data, we identified common and specific features of different senescent phenotypes. This research has the potential to pave the way for the development of new senotherapeutic drugs aimed at combating the negative consequences associated with the senescence process.

Video Abstract

**Supplementary Information:**

The online version contains supplementary material available at 10.1186/s12964-023-01280-4.

## Introduction

Senescence is the cellular response to endogenous and exogenous stressors, characterized by the loss of cell division, growth, and function [12]. Various stressors can induce cellular senescence, including genomic events such as telomere shortening, non-telomeric DNA damage, excessive mitogenic signals, as well as non-genotoxic stressors like perturbations to chromatin organization [11, 21]. Senescent cells exhibit numerous features, such as enlarged and flattened morphology, senescence-associated β-galactosidase activity, senescence-associated heterochromatin foci, altered gene expression, telomere-dysfunction-induced foci, DNA segments with chromatin alterations reinforcing senescence (DNA-Scars), and senescence-associated secretory phenotype (SASP) [12, 21, 44]. However, many of these characteristics are not specific to senescent cells to their heterogeneity. A clear-cut definition of senescent cells does not currently exist. Therefore, it is necessary to evaluate senescent cells using a combination of features. The development of a more universal marker for senescent cells is required. This can be a challenging endeavor as senescence is a constantly evolving process that cannot be examined as a fixed outcome. A crucial event in the transition from early to late senescence is the shift towards a more inflammatory phenotype, which can be attributed to the secretion of numerous cytokines by senescent cells [2, 7]. These cytokines, in turn, regulate various functions of the immune system. In this context, certain factors and biological features that may serve as hallmarks of early senescence stages may not necessarily be indicative of late and final senescence stages.

In our body, senescent cells not only contribute to organismal aging but also play a role in tissue development and wound healing. Moreover, they can serve as either inhibitors of cancer or facilitators of tumor growth [7, 11]. Senescent cells perform their diverse functions through the production of the SASP, whose molecules act as autocrine factors, reinforcing senescence in damaged cells. Furthermore, components of the SASP can function as paracrine or long-distance factors, promoting senescence in healthy cells that have not been directly affected by harmful stimuli [7, 17, 26].

A comprehensive analysis of the SASP would yield profound insights into the functionality of senescent cells. Previous studies have revealed that the components of SASP are influenced by the type of stressor, the elapsed time since the stress event, and the cell type involved [7, 17, 26]. For example, early senescent cells secrete SASP components that include various growth factors and cytokines, contributing to anti-tumorigenic effects, wound healing, and tissue development. As time progresses, senescent cells enter late senescence and modify the composition of SASP, leading to an enrichment of pro-inflammatory factors that promote cancer and aging [9]. Therefore, cellular senescence is a dynamic phenomenon, and analyzing a single time point is insufficient. A comprehensive analysis that takes into consideration the factors influencing these changes is necessary to fully comprehend SASP. Previous studies focused on the secretome have attempted to provide an overall understanding of the changes associated with senescence. However, the variations in stressors, senescence stages, and cell types used in these studies make it challenging to directly compare their conclusions. In this study, we conducted a meta-analysis of SASP protein lists collected from previous studies to elucidate the overall landscape of senescence. By employing gene ontology (GO) analysis and REACTOME analysis, our goal was to clarify the induction process of senescence and identify “true" key factors that could serve as universal markers for senescent cells. Enhancing our understanding of senescence may offer valuable insights for the development of novel senotherapeutic drugs aimed at mitigating the adverse effects of the senescence process.

## Results

### Study selection and characteristics

**Fig. 1:**
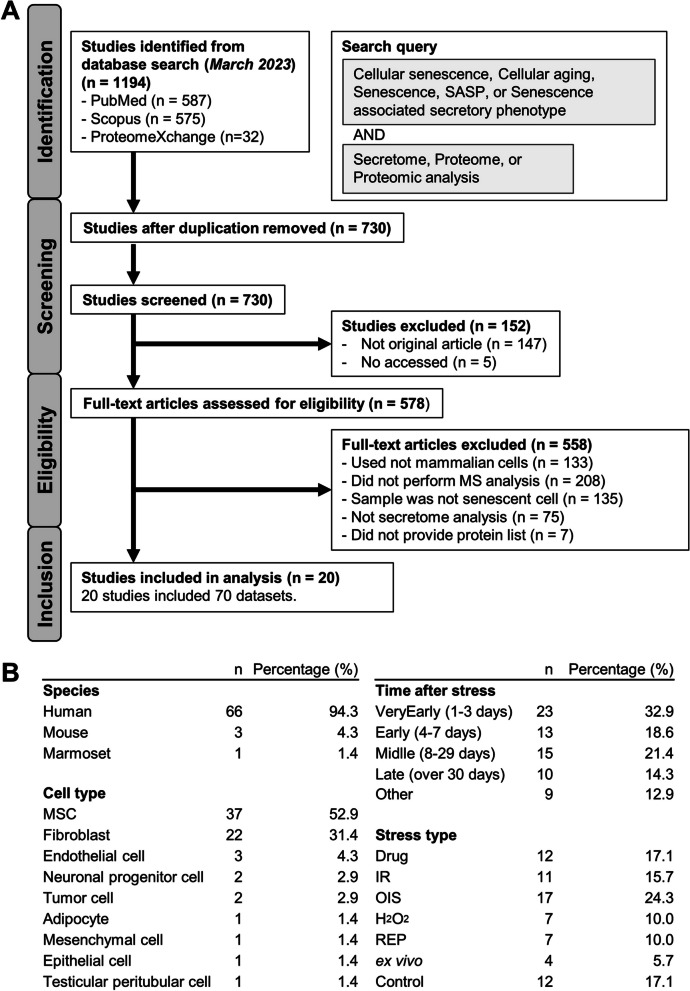
Overview of the data collection and information. **A** The flowchart illustrates the inclusion and exclusion process. **B** The characteristics of protein lists are presented, including species, cell type, elapsed time since stress, and stressor type

The study selection process and its characteristics were presented in Fig. 1A. A total of 1,194 research papers were identified from PubMed (*n* = 587), Scopus (*n* = 575), and ProteomeXchange (*n* = 32). After removing 464 duplicate studies and 152 non-original articles, 578 studies were considered as full texts for evaluation. Eventually, our analysis included 20 studies comprising 70 lists of proteins (Table S1). The details of the protein lists can be found in Fig. 1B. Out of these lists, 66 were derived from humans (94.3%), 3 from mice (4.3%), and 1 from marmosets (1.4%). In terms of cell type, more than 80% of the lists consisted of 37 mesenchymal stromal cell (MSC) lists (52.9%) and 22 fibroblast lists (31.4%). The elapsed time since stress was categorized into five classes: very early (1–3 days), early (4–7 days), middle (8–29 days), late (over 30 days), and other (mostly cells in a state of senescence from ex vivo experiments). Regarding the time course, the most frequently represented time point was very early (*n* = 23, 32.9%), while the least common was late (*n* = 10, 14.3%). The types of stressors were summarized as follows: oncogene-induced senescence (OIS) (*n* = 17, 24.3%), drug treatment (Drug) (*n* = 12, 17.1%), irradiation (IR) (*n* = 11, 15.7%), hydrogen peroxide treatment (H_2_O_2_) (*n* = 7, 10.0%), replicative stress (REP) (*n* = 7, 10.0%), originally senescent cells (ex vivo) (*n* = 4, 5.7%), and control (*n* = 12, 17.1%). OIS was the most common stressor type, while ex vivo was the least common. Due to the limited availability of data from mice and marmosets, only human-derived data were utilized for further analysis to avoid challenges associated with interpreting species differences.

### Common and specific components of SASP Protein Lists

**Fig. 2:**
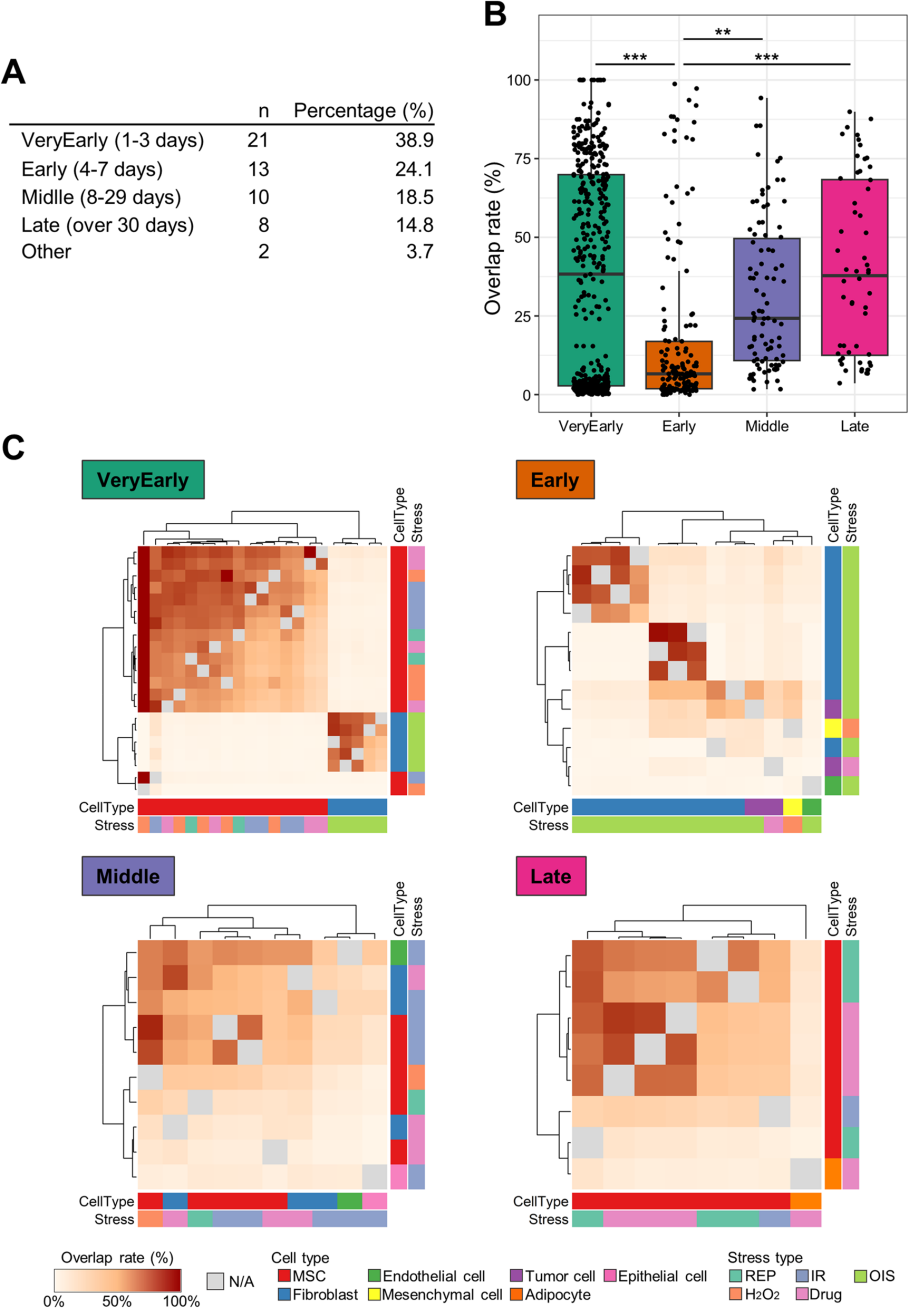
Similarity analysis at different time points since senescence induction. **A** The data are classified, and the number of protein lists obtained from senescent human cells is indicated. **B** A box plot displays the overlap rates at each time point. Statistical significance is denoted by asterisks (*p < 0.05, **p < 0.01, ***p < 0.001). **C** A heatmap depicts the overlap rates, with grey color indicating overlap rates calculated from the same protein lists. The color label positioned at the bottom and right side of the heatmap represents the characteristics of the data

Initially, our search focused on identifying common components of SASP using human data categorized into different time-series subgroups (Fig. 2A). We discovered five proteins (plasminogen activator inhibitor-1 (PAI-1), vimentin, galectin-1, insulin-like growth factor-binding protein (IGFBP) 4, and IGFBP7) that consistently appeared in the late stage. However, no common proteins were found at the other time points. To evaluate the similarity of SASP components across various protein lists, we utilized the "overlap rate" calculation, defined by the following formula: (Number of common proteins in protein list X and protein list Y) / (Total number of proteins in protein list X) × 100 = overlap rate of protein list X to protein list Y (%). The "overlap rate" signifies the percentage of proteins in protein list X that overlap with proteins in protein list Y. We computed the overlap rate for every possible combination of protein list within each time point and presented the results as overlap rates (Fig. 2B). Notably, the overlap rate during the early stage was significantly lower compared to the very early stage, while the overlap rates for the middle and late stages were significantly higher than that of the early stage (Fig. 2B).

To visually represent the overlap rates for each time point, we generated a heatmap (Fig. 2C). The heatmap displayed two distinct clusters in the very early stage and three clusters in the early stage. However, the middle and late stages exhibited a varied distribution without clear clusters. In order to further explore the factors contributing to the classification in the very early and early stages, we analyzed the percentage of cell types and stressor types (Figure S1). In the very early stage, we observed that fibroblasts were specifically assigned to cluster 2, alongside OIS. In the early stage, fibroblasts and OIS were present in both cluster 1 and cluster 2, and they were also found in cluster 3 along with other cell types and stressors (Figure S1). Notably, protein lists originating from the same cell type and stress source were occasionally assigned to different clusters, indicating the presence of additional factors influencing their classification.

**Fig. 3:**
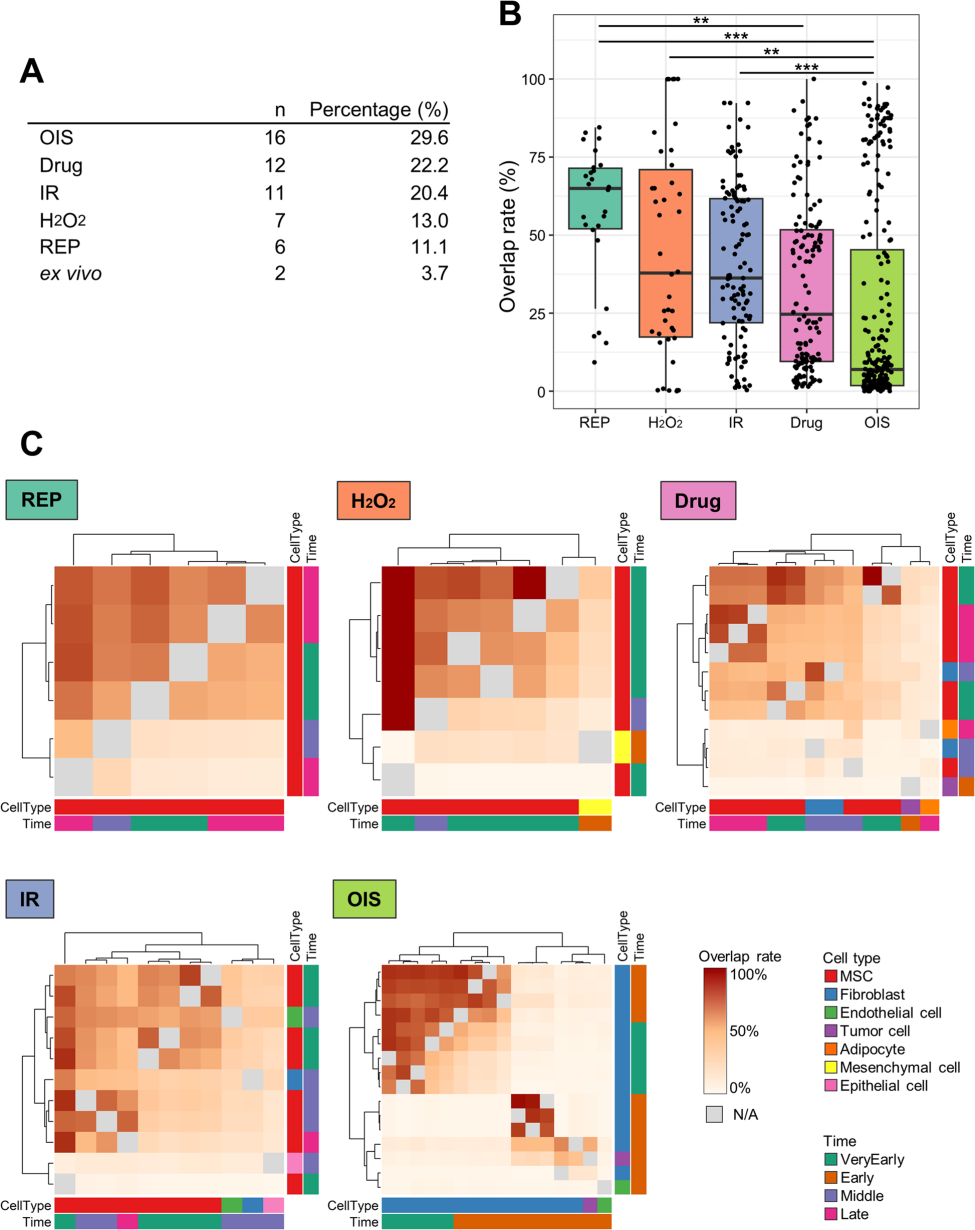
Similarity analysis among different stressor types. **A** The data are classified, and the number of protein lists obtained from senescent human cells is indicated. **B** A box plot displays the overlap rates for each stressor type. Statistical significance is denoted by asterisks (*p < 0.05, **p < 0.01, ***p < 0.001). **C** A heatmap depicts the overlap rates, with grey color indicating overlap rates calculated from the same protein lists. The color label positioned at the bottom and right side of the heatmap represents the characteristics of the data

Subsequently, we conducted a search for common components of SASP using human data grouped based on stressor type (Fig. 3A). Due to the limited availability of ex vivo samples, they were excluded from the analysis. Within the REP subgroup, we identified twenty-five proteins associated with the extracellular matrix (ECM) (collagen alpha-1(I) chain, collagen alpha-2(I) chain, collagen alpha-1(III) chain, collagen alpha-1(V) chain, collagen alpha-2(VI) chain, collagen alpha-3(VI) chain, periostin, decorin, fibrillin-1, fibronectin, thrombospondin-1, PAI-1, matrix metalloprotease(MMP)-2, metalloproteinase inhibitor-1), cytoskeleton (vimentin, annexin A2), growth factors (IGFBP4, IGFBP7, TGFBI, follistatin like 1), and others (heat shock protein family A member 5, protein-lysine 6-oxidase, peptidyl-prolyl cis–trans isomerase A (PPIA), testican-1, pentraxin 3) that were commonly observed. No common proteins were found in the other stressor subgroups. Similar to the analysis conducted among time points, we calculated the overlap rates for all combinations within each stressor type. The REP, H_2_O_2_, and IR subgroups exhibited significantly higher overlap rates compared to the OIS subgroup. Additionally, the REP subgroup had significantly higher overlap rates with the Drug subgroup (Fig. 3B). To visualize the overlap rates for each stressor type, we created a heatmap (Fig. 3C). The REP, H_2_O_2_, IR, and Drug subgroups displayed a heterogeneous distribution without forming specific clusters. Conversely, the OIS subgroup could be classified into three distinct clusters. To gain further insights, we examined the percentage of cell types and stressor types within each cluster of the OIS subgroup. Fibroblasts were exclusively present in clusters 1 and 2, while also appearing in cluster 3 along with other cell types (Figure S2). It is important to note that currently, there is no published data available on OIS in the middle and late conditions.

### Similarity analysis of SASP at different time points by GO and REACTOME

**Fig. 4:**
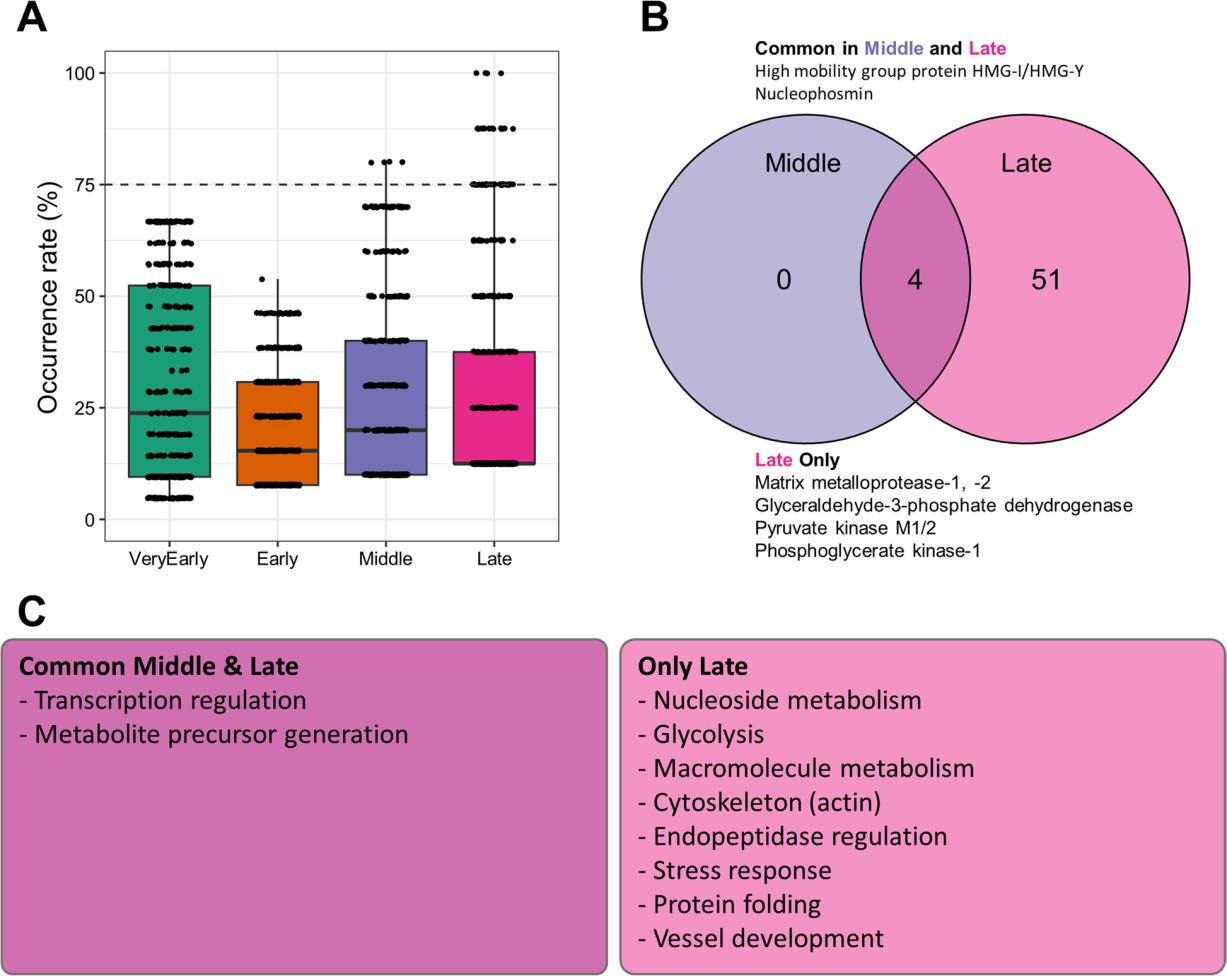
Gene ontology analysis of SASP classified by time series. **A** A box plot is presented to show the occurrence rates at each time point. The horizontal dashed line represents the threshold for selecting common ontology occurrence rates higher than 75%. **B** A Venn diagram is provided to identify the common and specific ontologies among the different time points. The names of proteins with central roles in each ontology and frequently included in datasets at each time point are indicated. **C** The main outcomes of the GO analysis are summarized. Common and specific ontologies are categorized based on their functions and displayed in the box

To elucidate the functional roles of SASP components at different time points, we conducted a GO analysis (Fig. 4). Significantly enriched ontologies were identified using a false discovery rate (FDR) threshold of 0.05 or lower. Occurrence rates were then calculated to determine the frequency of ontologies within the literature-retrieved lists for each time point. Notably, ontologies with a 100% occurrence rate were exclusively observed in the late stage (Fig. 4A). However, considering a 100% occurrence rate as the threshold proved to be too stringent. Therefore, we focused on ontologies present in at least 75% of the analyzed datasets with an FDR of less than 0.05, designating them as common ontologies.

Our analysis did not reveal any ontologies meeting the aforementioned criteria in the very early and early stages. However, in the middle stage, we identified four common ontologies that overlapped with those found in the late stage (Fig. 4B). These ontologies were associated with "transcription regulation" and "metabolites involved in energy production" (Fig. 4C). In the late stage, we detected 55 ontologies surpassing the 75% threshold. These ontologies included "Nucleoside metabolism," "Macromolecule metabolism," "Glycolysis," "Cytoskeleton," "Endopeptidase regulation," "Stress response," and "Protein folding" (Fig. 4C).

The ontologies identified in the late-stage of senescence suggest that senescent cells undergo metabolic adaptations to maintain their permanent condition. The observed alterations in cytoskeleton structures align with the acquired senescent phenotype. The regulation of endopeptidases, such as MMP1 and MMP2, corresponds to the substantial remodeling of the extracellular matrix occurring within the senescent cell microenvironment [32]. Additionally, certain enzymes, including glyceraldehyde-3-phosphate dehydrogenase (GAPDH), pyruvate kinase M1/2 (PKM), and phosphoglycerate kinase 1 (PGK1), were consistently detected in the late stage [13, 28].

The GO analysis revealed significant changes in biological processes following the onset and progression of senescence. To further investigate the function of SASP, we conducted an in-depth study using REACTOME analysis, which allows for the identification of significant signaling circuits in protein datasets. This pathway database considers any binding, activation, translocation, degradation, and other biochemical phenomena involving a catalyst as a 'reaction' among molecules. Similar to the GO analysis, we identified common pathways using FDR and occurrence rate.

**Fig. 5:**
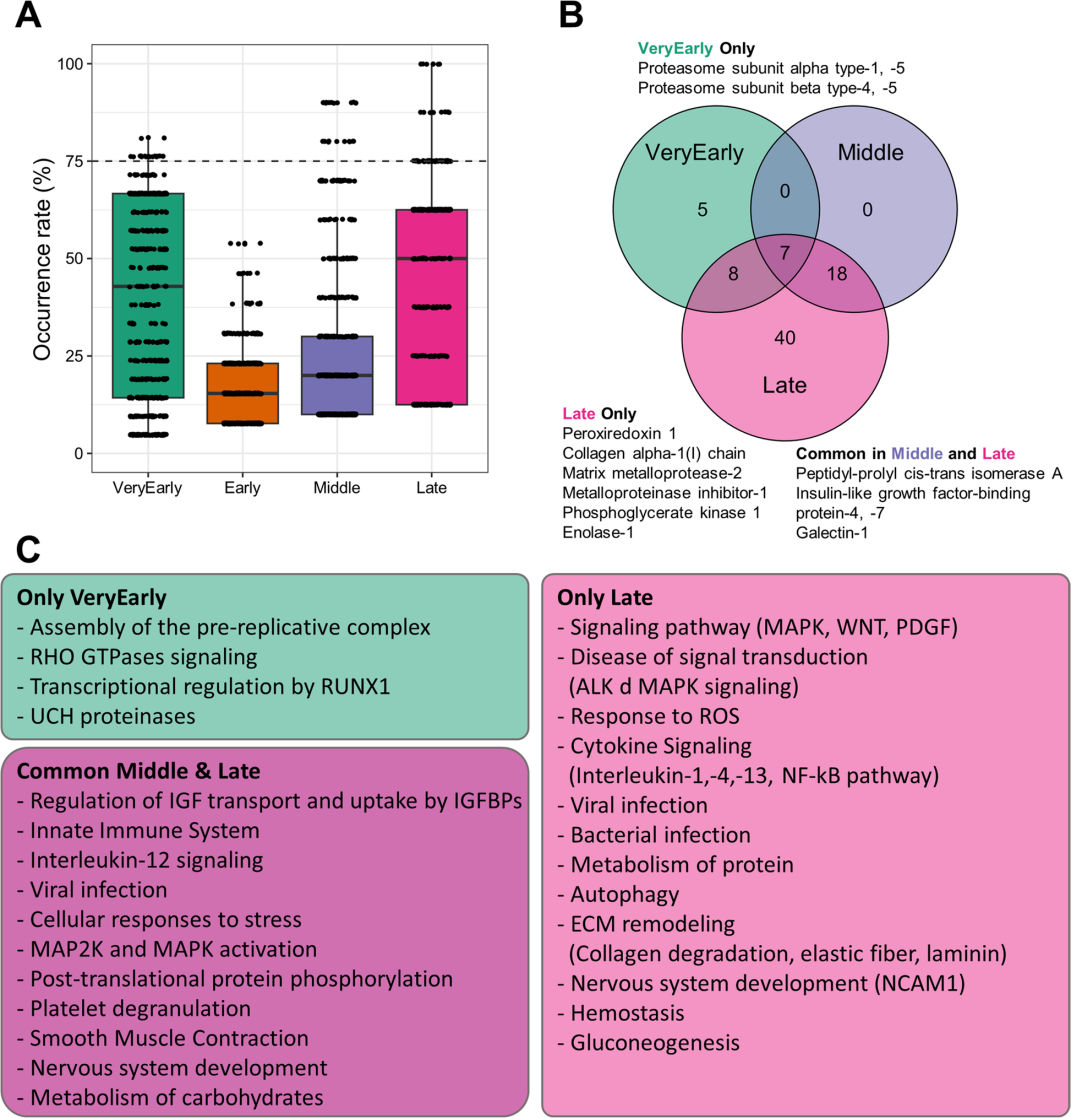
REACTOME analysis of SASP classified by time series. **A** A box plot is displayed to illustrate the occurrence rates at each time point. The horizontal dashed line represents the threshold for selecting common pathway occurrence rates higher than 75%. **B** A Venn diagram is utilized to identify the common and specific pathways among the different time points. The names of proteins with central roles in each ontology and frequently included in datasets at each time point are indicated. **C** The main outcomes of the REACTOME analysis are summarized. Common and specific pathways are categorized based on their functions and presented in the box

In the early stage, we did not find pathways present in more than 75% of the protein lists. However, we identified twenty pathways from the very early stage, twenty-five pathways from the middle stage, and seventy-three pathways from the late stage (Fig. 5A). The pathways identified in the middle stage were common to both the very early and late stages or exclusive to the late stage (Fig. 5B). No specific pathways were identified for the middle stage. However, we found specific pathways for the very early and late stages (Fig. 5B). The absence of pathways that were present in more than 75% of the early stage datasets may suggest that this time point represents the most heterogeneous stage of senescence progression.

The RUNX1 and UCH deubiquitinate pathways were identified as specific pathways in the very early stage (Fig. 5C). These pathways have been reported to be involved in the regulation of the proteasome, which plays a crucial role in senescence progression [4, 15]. In other words, our results suggest that RUNX1 and UCH may play a key role in triggering senescence. Proteasome 20S components, such as proteasome subunit alpha type (PSMA)1, PSMA4, PSMA5, proteasome subunit beta type (PSMB)-4, and PSMB5, were also consistently detected in most protein lists [14].

In the common pathways of the middle and late stages, we identified pathways related to IGF signaling ("Regulation of IGF transport and uptake by IGFBPs"), inflammation ("Innate immune system," "Interleukin-12 signaling," "Viral infection"), stress response ("Cellular responses to stress"), and metabolism ("Metabolism of carbohydrates") (Fig. 5C). Specifically, the PPIA protein was found to be a common component in pathways related to inflammation [35]. PPIA is important for protein folding and aging and is also associated with the proteasome. Additionally, pathways related to "Cellular response to stress" included commonly assigned proteins such as HSPs and PRDXs. HSPs and PRDXs contribute to senescence stress resistance by regulating protein folding and preventing oxidation [20, 46].

The pathways specifically found in the late stage were also involved in regulating biological functions identified in both the middle and late stages. These pathways include those responsible for i) stress response ("Response to ROS"), ii) inflammation ("Cytokine signaling," "Viral infection," and "Bacterial infection"), and iii) carbohydrate metabolism ("Gluconeogenesis") (Fig. 5C). In addition to these pathways, the late-stage SASP specifically contained proteins related to the autophagy pathway and ECM remodeling pathway.

### Similarity analysis of SASP induced by different stressors

**Fig. 6:**
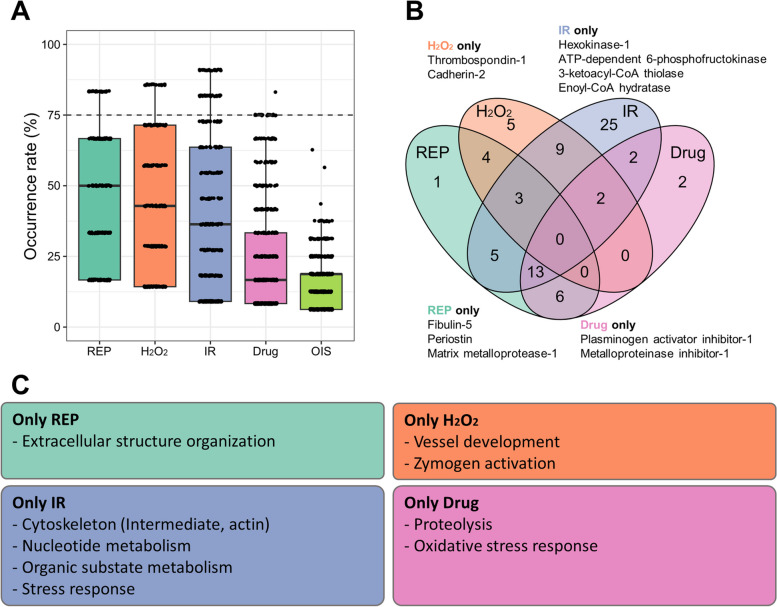
Gene ontology analysis of SASP classified by stressor type. **A** A box plot is presented to display the occurrence rates for each stressor type. The horizontal dashed line represents the threshold for selecting common ontology occurrence rates higher than 75%. **B** A Venn diagram is utilized to identify the common and specific ontologies among the different stressor types. The names of proteins with central roles in each ontology and frequently included in datasets at each time point are indicated. **C** The main outcomes of the GO analysis are summarized. Common and specific ontologies are categorized based on their functions and presented in the box

We conducted a subgroup analysis to explore the relationship between SASP components and different types of stressors. The threshold used to determine statistical significance for enriched ontologies and pathways was the same as that used in the subgroup analysis of the different time points. None of the ontologies for OIS were present in more than 75% of the protein lists (Fig. 6A). The absence of pathways that were present in more than 75% of OIS datasets suggests that this stressor produces highly heterogeneous outcomes, as we have already observed in the heatmap classification of SASP components.

In our analysis, we identified 59 ontologies in IR, 32 in REP, 23 in H_2_O_2_, and 25 in Drug stressors (Fig. 6B). There were no common ontologies among these stressors (Fig. 6B). A comprehensive analysis of ontologies specifically present in each stressor subgroup yielded incongruous results. For example, the ontology named "Extracellular structure organization" was identified only in the REP data list, even though it is well known that ECM remodeling occurs in senescence regardless of the specific stressor [32]. Similarly, the "Oxidative stress response" ontology was specifically identified in the Drug data list, despite the fact that the presence of reactive oxygen species (ROS) is a common feature of senescent cells [38, 41]. These peculiar results may be attributed to the limitations of the study (see Discussion).

It is important to emphasize that the presence of an ontology only in a specific subgroup does not exclude the possibility that some components of that ontology could also be present in other subgroups. In other words, the specific identification of the "Oxidative stress response" ontology in the Drug data list may indicate that this pathway plays a more prominent role for this particular stressor compared to the others.

**Fig. 7:**
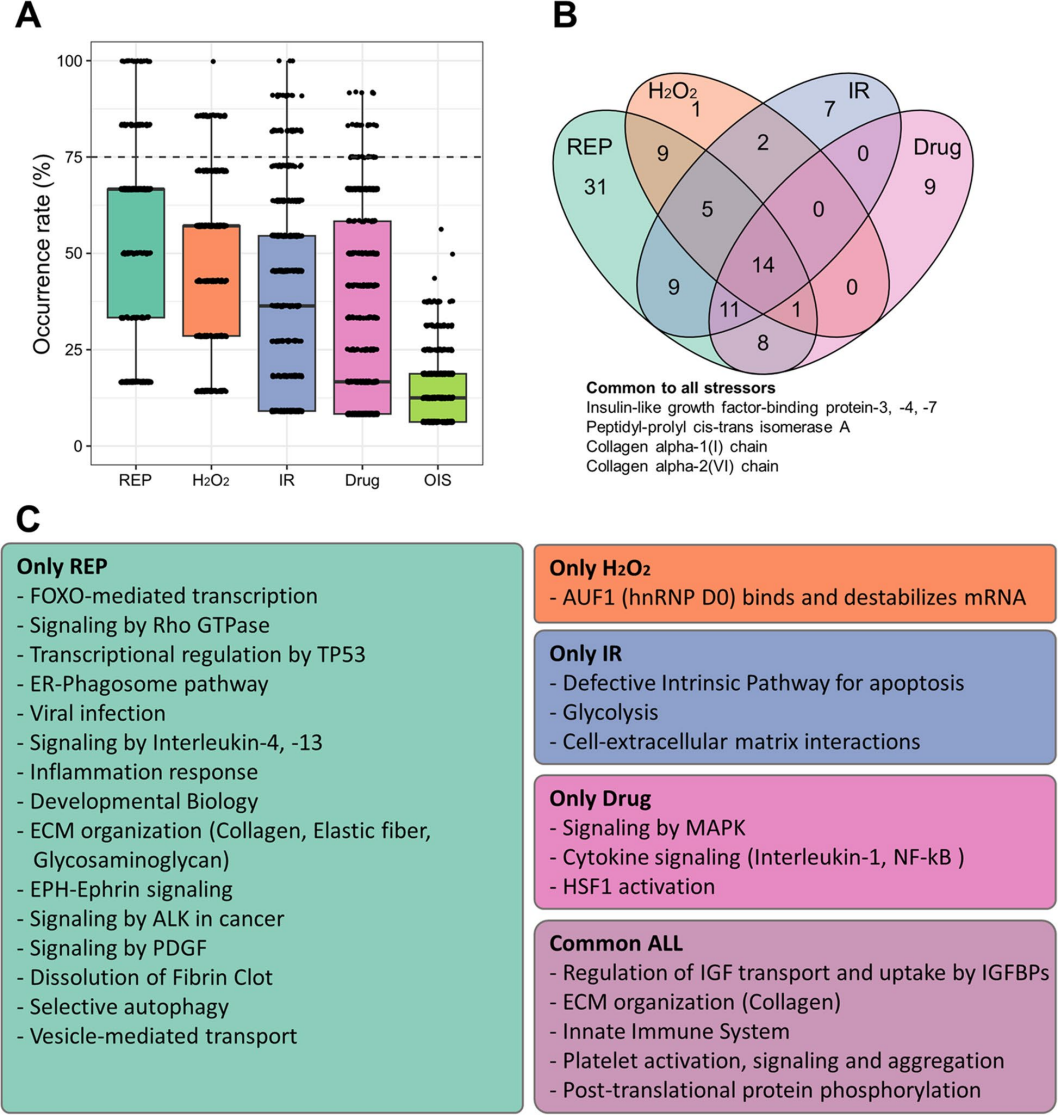
REACTOME analysis of SASP classified by stressor type. **A** A box plot is presented to illustrate the occurrence rates for each stressor type. The horizontal dashed line represents the threshold for selecting common pathway occurrence rates higher than 75%. **B** A Venn diagram is used to identify the common and specific pathways among the different stressor types. The names of proteins with central roles in each ontology and frequently included in datasets at each time point are indicated. **C** The main outcomes of the REACTOME analysis are summarized. Common and specific pathways are categorized based on their functions and presented in the box

We performed REACTOME analysis to examine the effect of stressor types on SASP in more detail (Fig. 7). Unlike the other conditions, we did not observe pathways in OIS that were present in more than 75% of the protein lists (Fig. 7A). For each stressor, we identified 88 pathways from REP, 32 pathways from H_2_O_2_, 48 pathways from IR, and 43 pathways from Drug. Out of these, 14 pathways were common to all stressors (Fig. 7B). These common pathways were associated with IGF signaling, ECM remodeling, and inflammation (Fig. 7C). This suggests that these three signaling circuits play a role in shaping certain common aspects of the senescent phenotype, regardless of the specific stressor.

Additionally, we identified pathways that were characteristic of each stressor type (Fig. 7C). For example, pathways involved in inflammation were found in multiple stressor types. Upon closer examination of the pathways, we discovered distinct signaling pathways with similar functions. For instance, REP exhibited interleukin (IL)-4 and -13 signaling, while Drug showed IL-1 and NF-kB signaling. This analysis allowed us to identify trends in the impact of each stress type on SASPs. Moreover, certain pathways were unique to specific stressors.

It should be noted that the presence of pathways, which refer to common activities of senescent cells, only in a specific subgroup does not imply their absence in the other conditions. It could be hypothesized that in the other conditions, the stress response may be less prominent and therefore not detectable in more than 75% of the analyzed data, as observed in the GO analysis.

## Discussion

The onset of the senescent phenotype is characterized the secretion of many factors (SASP), which can have positive effects on our bodies, such as anti-cancer properties, wound healing, and contributions to tissue development. These effects are attributed to the SASP produced by senescent cells in their earlier stages. However, senescence can also promote cancer and aging, primarily due to the late stage SASP.

Senescence is a dynamic process influenced by a variety of factors, which greatly affects SASP composition and functions. However, to our knowledge, there is no comprehensive secretome analysis that considers these influences. In this study, we performed a meta-analysis of 70 protein lists from 20 studies to clarify the induction process of senescence. The analysis revealed the following points: I) The IGF and IGFBP signaling pathways are representative common factors in senescent cells. II) The RUNX1 and UCH deubiquitination that regulate of proteasome activity were enriched at the very early stage (1–3 days). III) SASP of the middle stage and late stage were enriched inflammatory pathway related protein, including IL-1, -4, -12, -13, and NF-kb. IV) There is a change in carbohydrate metabolism towards glycolysis during senescence induction. V) Senescent fibroblasts induced by OIS were found to be distinct from other senescent cells.

### SASP Similarity among time course and stressor type

In the time course analysis, the lowest similarity between SASPs was observed at the early stage (4–7 days). Senescence can be triggered by various stressors, and the differences in stress intensity, along with random damage to DNA and other macromolecules, can lead to heterogeneous initiation of the senescence process. This variability at the early stage and the classification of data into three clusters can be attributed to these factors.

At the middle stage (8–29 days), the absence of specific ontologies and pathways suggests that this stage represents a genuine transition phase from the initial stage of senescence to the final senescent state. As time progresses, the similarity of SASP components increases. Some common proteins (PAI-1, vimentin, galectin-1, IGFBP4, and IGFBP7) were only detected at the late stage and not in other stages. These proteins may be essential for survival and expansion in late senescent cells, or they may represent common phenotypic traits. This indicates that, given enough time, senescent cells may exhibit a more universal state regardless of the initial conditions. However, it also emphasizes the importance of considering the elapsed time since the induction of stress when designing experiments and interpreting results. Heterogeneity at early senescence and hight simirality at late senescence can be exprained by the necessary changes at each stage of senescence induction. In early senescence, a rapid change from the original cellular state to senescent cells occurs. The change is dependent on the initial state, and thus the composition of SASP differs from cell to cell and from stress to stress. In the middle and late stages, similar functional changes occur, such as metabolic changes for survival of senescent cells, modification of the microenvironment with changes in the ECM, and regulation of the immune system. This may be the reason for the differences in the proteins contained in the timepoints.

It should be noted that senescent fibroblasts induced by OIS may not serve as representative model of senescent cells, as they possess specific features and exhibit higher heterogeneity, especially in the early stage. These findings align with the results obtained from the analysis based on time points, where we observed that the protein content of SASP induced by OIS was distributed across three clusters, while in the other stressors, it was grouped into a single cluster. Therefore, caution should be exercised when comparing research results on senescence using the OIS model with studies involving other cell types or stress sources.

### Function of IGF and IGFBP signaling in senescence

IGFBPs have been extensively linked to senescent cells, particularly IGFBP3, 4, 5, and 7, which are known to be upregulated in senescent cells. Studies have demonstrated that these IGFBPs can induce senescence at both the cellular and individual levels when administered to either the culture medium or living organisms [3, 16, 25, 40]. In our analysis, we consistently detected IGFBP4 and IGFBP7 in all datasets at the late stage. Furthermore, the IGF and IGFBP signaling pathway emerged as a common pathway in both the middle and late stages, irrespective of the type of stressor. These findings align with previous analyses and underscore the significant role of the IGF and IGFBP pathway in senescence. They suggest that factors associated with this pathway could serve as a universal marker for identifying senescent cells.

### Protein metabolism in senescent cells

The downregulation of proteasome expression and its impaired ability to process damaged proteins are crucial for initiating senescence induction [13, 14, 30]. In this study, we observed the involvement of the UCH deubiquitination and RUNX1 signaling pathways, which regulate proteasome activity, in SASP during the very early stage (1–3 days). These findings suggest that UCH and RUNX1 may play a role in modulating proteasome activity during this early stage of senescence. Notably, we also detected the presence of 20S proteasome subunits as components of SASP. The 20S proteasome is found in over 25 different body fluids and has been associated with the activity of certain diseases [6, 31]. However, the specific function of these proteasome subunits in SASP remains unclear. The increased levels of 20S proteasome within SASP present a promising avenue for further investigation in senescence research. Autophagy, which is involved in intracellular protein metabolism, has been reported to both promote and inhibit senescent progression [8, 39]. The identification of an autophagy-related pathway at the late stage aligns with previous research and underscores the significance of autophagy in the survival of late senescent cells.

### Carbohydrate metabolism changes during senescence progression

The carbohydrate metabolism pathway, specifically glycolysis, is identified during the late stage, and it is considered the preferred metabolic pathway in senescent cells [1, 13]. This is believed to correspond to the increased demand for proteins and lipids required for senescence-associated events, including the secretion of factors known as SASP and cellular enlargement [45]. Glycolysis is also responsible for elevated lactate production through pyruvate synthesis, involving upregulated lactate dehydrogenase (LDHA), PKM, serinolysis, and glutaminolysis. PKM facilitates the final conversion step of glycolysis, leading to pyruvate production [13]. These findings underscore the importance of metabolic changes towards glycolysis for the survival of senescent cells and the manifestation of a senescent phenotype.

### Inflammation and immunology factors including SASP

When senescent cells are present in the body, they are typically eliminated by immune cells once they have surpassed the early senescence stage, which promotes tissue repair. However, certain senescent cells secrete immune-related substances that enable them to evade immune cell removal and persist until the late senescence stage. This persistence can have negative effects, such as promoting aging and cancer development. To comprehend the survival mechanisms of these senescent cells, it is important to focus on the cytokines they secrete.

In this study, the IL-12 pathway was identified as a common circuit in SASP from middle and late-stage senescent cells. Additionally, signaling associated with IL-1, -4, and -13 was found to be specific to SASP from late-stage senescent cells. The presence of IL-12-associated pathways prior to other inflammation-related signaling circuits aligns with its role in initial inflammatory events. During early inflammation, IL-12 is produced and plays a crucial role in promoting the differentiation of naive T cells into Th1 cells [22]. It is recognized as a factor that activates T cells, enhancing their growth and functionality. IL-12 stimulates the synthesis of interferon-gamma (IFN-γ) and tumor necrosis factor-alpha (TNF-α) by T cells and natural killer (NK) cells. Furthermore, it counteracts the inhibitory effects of IL-4 on IFN-γ production [47]. This role coincides with the presence of other inflammation pathways identified in late SASP, suggesting a gradual accumulation of pro-inflammatory factors in SASP.

Notably, IL-13-related signaling was identified in late-stage SASP. IL-4 and -13 are produced by Th2 cells and may serve as modulators of inflammation, potentially possessing anti-inflammatory activity [24]. A comprehensive analysis of pro- and anti-inflammatory factors present in SASP extends beyond the scope of this study. Nevertheless, it is possible to hypothesize that late senescence SASP, in addition to its well-known pro-inflammatory components, may contain anti-inflammatory molecules that balance and modulate the activity of pro-inflammatory factors. Further investigation is warranted to explore this aspect.

### Potential targets of senotherapeutic drugs

The senotherapeutic drugs are classified into senolytics drug, selectively kill senescent cells or senomorphics drugs, suppress the SASP that affects negative effects with aging [34]. The present analysis disclosed IGFBP was a representative common component of SASP, and previous studies reported that IGFBP alone can induce senescence. It is presumed that IGFBP plays an important role in the propagation of senescence by SASP. Therefore, the use of senomorphic drugs targeting IGFBP and IGF receptors may be able to prevent the progression via SASP of aging throughout the body. We have also found that UCH deubiquitination and RUNX1 signaling pathways have some role in the early stages of senescence induction. If this pathway can be regulated to normalize proteasome function and eliminate abnormal proteins involved in the development of senescent induction, it may lead to the development of senotherapeutic drugs based on a new approach to suppress senescent cell development itself. The data in this study provide valuable information for the development of senotherapeutic drugs, particularly senomorphic drugs. In addition, this approach is novel and has the potential to become a new tool to bridge aging research and clinical practice.

### Limitations of this study

The research has certain inherent limitations. I) Data acquisition and analysis methods vary among the studies included. Unlike transcriptome analysis, there is no standardized method for integrating and correlating quantitative proteomic data. To mitigate potential bias, we integrated the proteomic data solely as a protein list without incorporating quantitative information. II) We used datasets with various backgrounds (e.g. different proteomics analysis methods, cell type, stress type, and timepoint). Therefore, we could not able to determine whether the differences among protein lists originated from the technical problem or biological differences. That’s why we focused on the common protein and pathways to obtain more reliable conclusions. III) Generally speaking, several authors have classified the stages of senescence into early, full, and deep (late) senescence by considering the elapsed time since stress induction [43]. It is well known that the final stage of senescence, at least in the in vitro models, is followed by cell death [36]. This cell death may occur either a few days following stress or at a later time. Hence, the division of senescence into four distinct stages and their classification is somewhat arbitrary. The purpose was to identify key milestones in the continuous and progressive process of senescence. Indeed, early and late stages need to be defined solely by the elapsed time period from stress induction. Moreover IV) Not all stressor types are uniformly represented at all time points. For instance, senescent cells induced by replicative stress (REP) are more prevalent in the late stage, while senescent cells induced by oncogene-induced senescence (OIS) are more common in the early stage. Consequently, due to the limited sample size, it was not feasible to analyze the combined effects of these factors or assess cross-effects comprehensively.

V) It could be of interest to integrate SASP studies performed with MS analysis with others, mainly performed with cytokine arrays and ELISA assays. MS analysis is an unbiased analysis method used to examine the SASP without any previous hypothesis. On the other hand, assays based on cytokine array or ELISA analysis are based on specific hypotheses, where a predetermined set of factors that might be present in the SASP is selected. For instance, cytokine arrays can target inflammation factors or factors involved in cancer, among others. Due to this fundamental difference in approach, direct comparisons between MS analysis and array/ELISA assays may not be appropriate.

It is worth noting that while there are established databases for storing MS (Mass Spectrometry) data, there is currently no equivalent repository for cytokine arrays. This can make it challenging to extract relevant data from published literature for assays.

Despite these limitations, we performed a PubMed analysis using the keywords "senescence" in the title and "SASP" in "title/abstract" and looked for SASP analysis by cytokine arrays. In several publications, as evidenced in the current study, we found that inflammatory interleukins (IL-1, IL-6, IL-8, IL-23, TNFs), ECM remodelers (metalloproteases), and IGF-signaling factors (IGFBPs) were among the most common SASP factors, with some peculiarities related to stressor type and time course [5, 10, 23, 27, 29, 37].

## Conclusions

By utilizing Gene Ontology and Network analysis on the comprehensive data we retrieved from a detailed meta-analysis, we successfully uncovered shared and distinct characteristics among various senescent phenotypes. This investigation holds promising prospects for advancing the field of senotherapy by facilitating the creation of novel drugs targeting the detrimental effects linked to the aging process.

## Material and method

### Inclusion criteria

Studies showing the following criteria were considered for inclusion in our analysis: 1) publication in a peer-reviewed journal, 2) utilization of mammalian cells in the study, 3) implementation of comprehensive proteome analysis through mass spectrometry with reported original data, 4) focus on secretome analysis of senescent cells, and 5) provision of a comprehensive protein list.

### Search strategy

In March 2023, we conducted a search to identify relevant studies and data from PubMed, Scopus, and ProteomeXchange. The following keyword was applied across all databases: (("cellular senescence" OR "cellular aging" OR “senescence” OR “sasp” OR "senescence associated secretory phenotype") AND (“secretome” OR “proteome” OR "proteomic analysis"). The searches were conducted on the titles and abstracts of articles published in English.

### Data collection

We carefully reviewed the search results to select articles that met our inclusion criteria and assessed their eligibility for the present study. Protein lists were extracted from the final selected studies. For quantitative data, we included proteins that were detected exclusively in senescent cells and showed significant enrichment within those cells. For qualitative data, we included all proteins detected in senescent cells. The protein lists were collected using UniProt IDs. In cases where the protein lists were initially provided in a different format, we converted them into UniProt IDs using the UniProt ID mapping tool. Isoforms were represented by appending a dash and a number. For example, Q9D0E1 and Q9D0E1-2 were not distinguished from each other. If different UniProt IDs referred to the same protein, we unified them into UniProt/Swiss-Prot IDs. Additionally, we collected experimental data such as species, cell type, stress type, and time since the induction of stress, in addition to the protein lists.

### Subgroup analysis

We conducted a subgroup analysis, specifically focusing on the elapsed time since stress, stressor type, and cell type. To evaluate the continuous variable of time, we divided it into five distinct time points: very early (1 to 3 days), early (4 to 7 days), middle (8 to 29 days), late (after 30 days), and other (originally senescent cells). Stressor types were categorized as follows: replicative stress (REP), hydrogen peroxide treatment (H_2_O_2_), irradiation (IR), drug treatment (Drug), oncogene-induced senescence (OIS), and senescent cells derived from animals or patients (ex vivo).

### Protein list similarity analysis

To assess the similarity of protein lists, overlap rates were calculated using the following formula: (Number of common proteins in protein list X and protein list Y) / (Total number of proteins in protein list X) × 100 = overlap rate of protein list X to protein list Y (%). A heatmap depicting the overlap rates was generated using the "ComplexHeatmap" package in R [19]. The protein lists were clustered based on Euclidean distance using the complete method. In the heatmaps, overlap rates calculated from the same datasets were displayed in grey.

### Gene ontology analysis

GO analysis was performed using PANTHER (http://www.pantherdb.org) with the following parameters: analysis type "PANTHER overrepresentation test," reference list "Homo sapiens,"annotation dataset "PANTHER GO-Slim Biological Process," test type "Fisher's exact," and correction method "Calculate False Discovery Rate" [33, 42]. The GO analysis was conducted for each dataset, and an ontology was deemed significant if it had a false discovery rate (FDR) of less than 0.05.

To identify common ontologies, we calculated the occurrence rate of each ontology within each subgroup. The occurrence rate represents the frequency at which an ontology appears within a subgroup. An ontology with an occurrence rate higher than 75% was considered a common ontology. The Venn diagram illustrating the overlapping ontologies was generated using the "ggvenn" package in R. Finally, ontologies with similar functions were summarized and categorized.

### REACTOME analysis

We conducted REACTOME analysis using the REACTOME database (https://REACTOME.org) [18]. An overrepresentation test was performed on each dataset to identify enriched pathways. Pathways with a false discovery rate (FDR) of less than 0.05 were considered statistically significant. Similar to the GO analysis, we calculated the occurrence rate of each pathway within subgroups. A pathway with an occurrence rate exceeding 75% was considered a common pathway. Finally, pathways with similar functions were summarized based on their annotations and biological relevance.

### Statistical analysis

One-way factorial ANOVA and Tukey's Honestly Significant Difference test were employed to assess the significance of differences among overlap rates across each subgroup. A *p*-value of less than 0.05 was considered statistically significant. To account for multiple testing, the False Discovery Rate (FDR) correction was applied. The computational analyses were performed using the R programming environment (version 4.2.2) and JMP Pro 17 software (SAS Institute, Cary, NC, USA).

### Supplementary Information

**Additional file 1: Table S1. **Detailed information of 20 studeis and 70 protein lists included in this study.

**Additional file 2: Figure S1.** Similarity analysis at very early and early stage of senescence. The percentage of each cell type and each stressor contributing to the classification in the very early and early stages is displayed. The heatmaps shown in Figure 2C are presented again. The composition of each cluster, categorized by cell types and stressors, is reported on the right side of the heatmaps.

**Additional file 3: Figure S2.** Similarity analysis of OIS. The percentage of each cell type and each time point contributing to the classification in the OIS is displayed. The OIS heatmap shown in Figure 3C is presented again. The composition of each cluster, categorized by cell types and time, is reported on the right side of the heatmap.
