# Supplementary figures and images for: Meta-analysis of senescent cell secretomes to identify common and specific features of the different senescent phenotypes: a tool for developing new senotherapeutics
Source: Cell Commun Signal. 2023 Sep 28;21:262. doi: 10.1186/s12964-023-01280-4 (PMC10537976; doi:10.1186/s12964-023-01280-4)

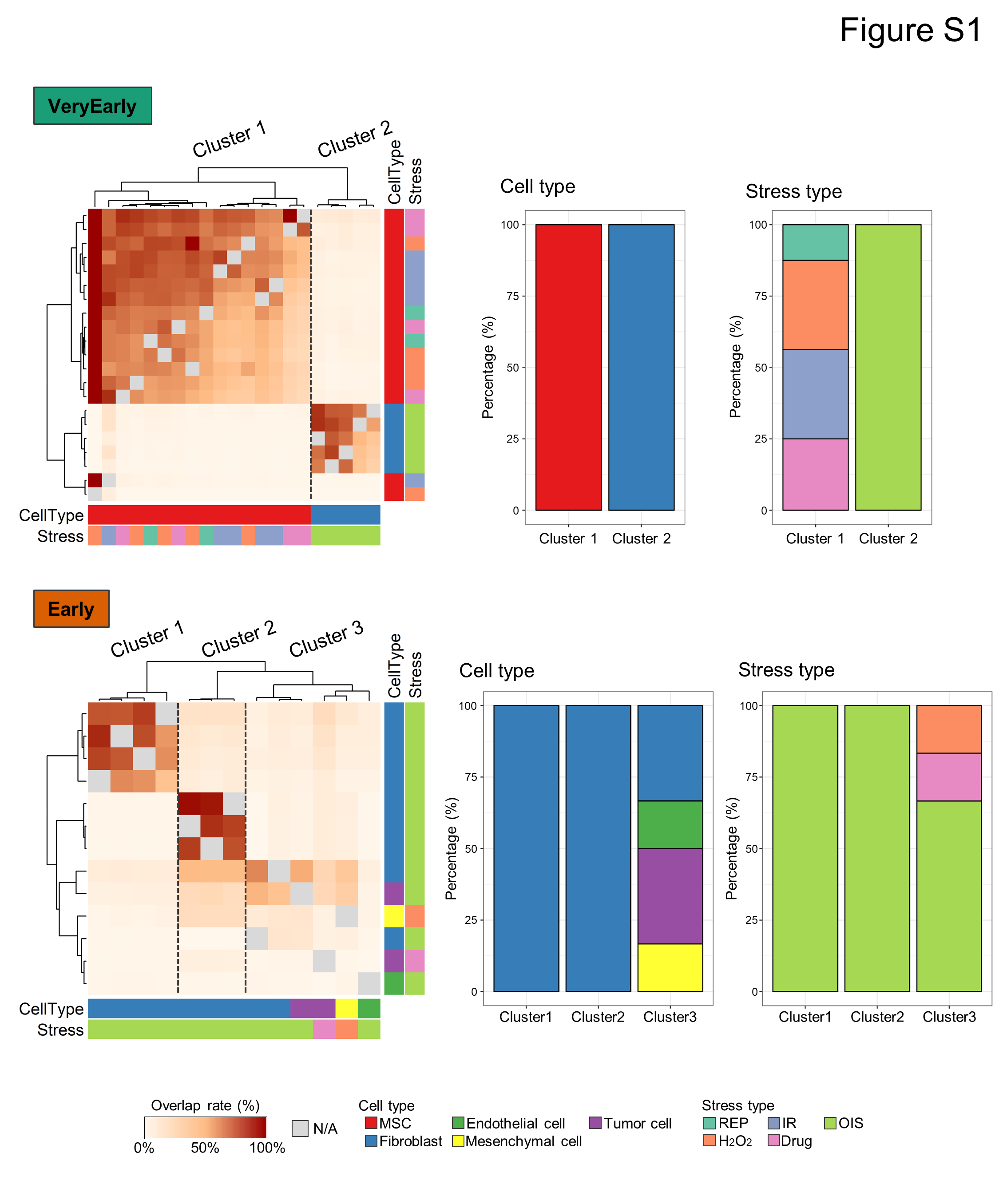

Supplement: Supplementary file 3 — Additional file 2: Figure S1. Similarity analysis at very early and early stage of senescence. The percentage of each cell type and each stressor contributing to the classification in the very early and early stages is displayed. The heatmaps shown in Figure 2C are presented again. The composition of each cluster, categorized by cell types and stressors, is reported on the right side of the heatmaps. [file 12964_2023_1280_MOESM2_ESM.tif]

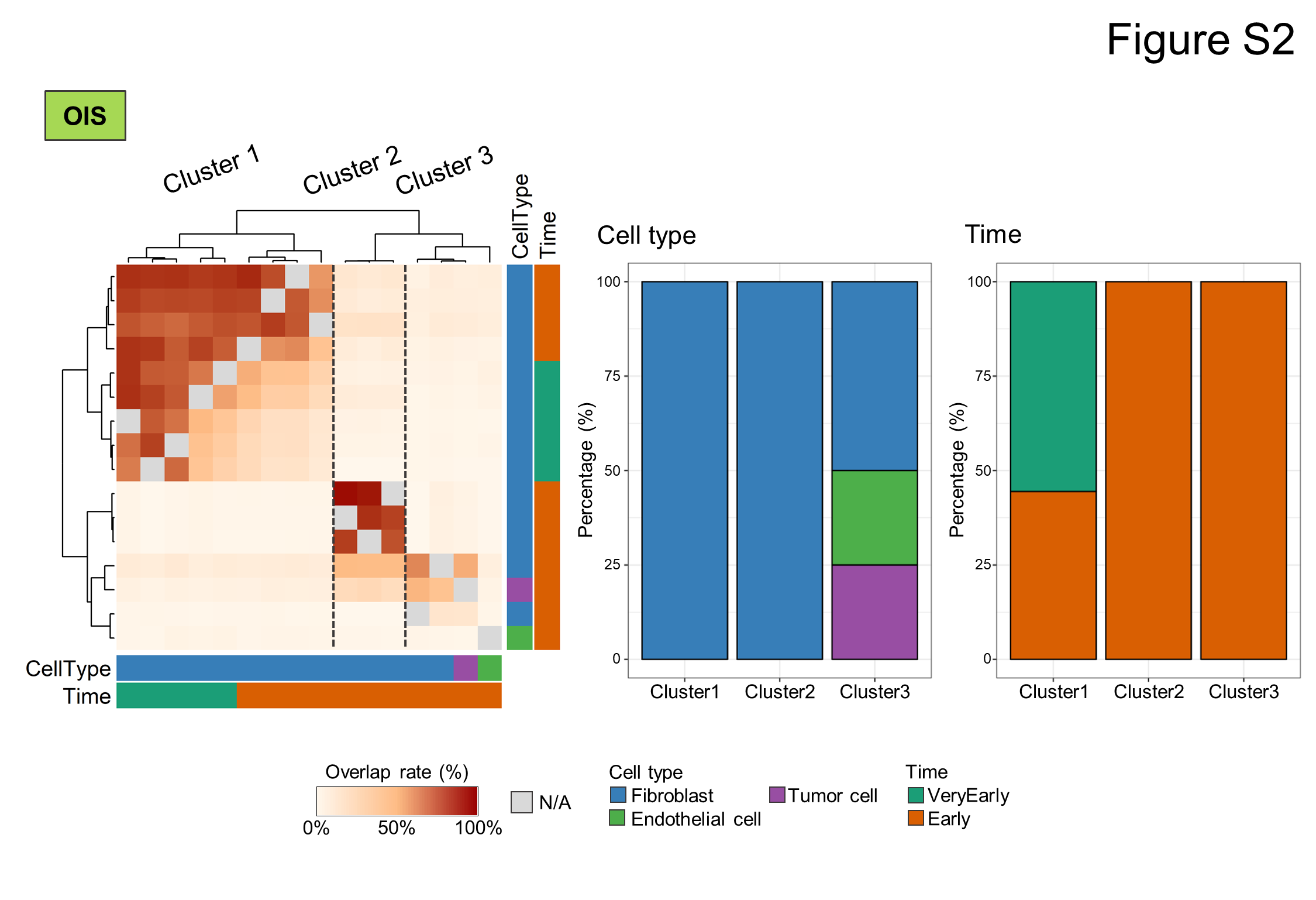

Supplement: Supplementary file 4 — Additional file 3: Figure S2. Similarity analysis of OIS. The percentage of each cell type and each time point contributing to the classification in the OIS is displayed. The OIS heatmap shown in Figure 3C is presented again. The composition of each cluster, categorized by cell types and time, is reported on the right side of the heatmap. [file 12964_2023_1280_MOESM3_ESM.tif]
